# Health related quality of life of HIV/AIDS patients on highly active anti-retroviral therapy at a university referral hospital in Ethiopia

**DOI:** 10.1186/s12913-017-2714-1

**Published:** 2017-11-15

**Authors:** Abdrrahman Shemsu Surur, Fitsum Sebsibe Teni, Wondwessen Wale, Yihenew Ayalew, Betel Tesfaye

**Affiliations:** 1 Department of Pharmaceutical Chemistry, School of Pharmacy, College of Medicine and Health Sciences, University of Gondar, Gondar, Ethiopia; 2 Department of Pharmaceutics and Social Pharmacy, School of Pharmacy, College of Health Sciences, Addis Ababa University, Addis Ababa, Ethiopia

**Keywords:** Health related quality of life, Patients living with HIV/AIDS, HAART, WHOQOL-HIV BREF

## Abstract

**Background:**

Highly active antiretroviral therapy improves the longevity of patients living with HIV/AIDS. We conducted the study in order to assess health related quality of life of HIV/AIDS patients and the association of socio-demographic and disease related variables with health related quality of life.

**Methods:**

Health facility based cross-sectional study among 400 HIV/AIDS patients taking highly active anti-retroviral therapy from Gondar University referral hospital was conducted. A pre-tested semi-structured questionnaire, which was adopted from World Health Organization Quality of life brief instrument, was used. The data were then analyzed using SPSS version 20 software for Windows.

**Results:**

The majority of the respondents reported to to have a good physical health (15.55). The World Health Organization clinical stage was found to be significantly associated with all the domains of health related quality of life. The current acute illness condition of the respondents, however, did not show significant association with any of the domains of health related quality of life.

**Conclusions:**

The six domains of health related quality of life were found to be moderate. The physical health and spirituality of the patients were relatively higher than their social relationship. Sex, age, educational status, residence and marital status showed significant association with at least one domain of health related quality of life.

## Background

Since the beginning of the epidemic, about 39 million people have died of HIV/AIDS. Globally, 35.0 million people were living with HIV at the end of 2013. Sub-Saharan Africa remains most severely affected with nearly 1 in every 20 adults living with HIV [1]. Moreover, the alarming increase of HIV/AIDS, inability to afford highly active anti-retroviral therapy (HAART), disability, stigma, loss of productivity due to illness, and chronic nature of the disease has made HIV/ AIDS one of the most important public health problems in sub-Saharan Africa countries.

People living with HIV/AIDS (PLWHA) show different symptoms which may involve flu-like symptoms like fever or rash occurring for a month or two after infection. Chronic diarrhea, rapid weight loss and other opportunistic infections and infection related cancers start appearing after many years [2]. In terms of mental health, in comparison with the general population PLWHA may be more likely to develop to develop mental disorders like depression or anxiety and major depression is the commonest psychiatric problem occurring associated to the disease [3, 4].

HIV/AIDS has also been associated in a complex way with impoverished housing conditions. The challenges of slums in urban areas like basic services, inadequate water, sanitation, overcrowding and others are worsened by the impact of HIV/AIDS. Problems like inadequate water and sanitation increase the disease burden [5]. Reports have also indicated that homelessness and unstable housing have been associated with greater HIV risk, to poor health outcomes and early death among PLWHA [6].

HAART has shifted the perception of HIV/AIDS from a fatal to a chronic and potentially manageable disease. The use of HAART has become the cornerstone of the clinical intervention to prevent transmission and slow progression of HIV infection in individuals living with HIV/AIDS [7]. However the medications are associated with adverse effects which may contribute to decreased adherence to regimens. These included bone density events, dyslipidemia, GI effects, hepatic effects, diabetes mellitus/ insulin resistance, Stevens-Johnsons Syndrome, renal effects, rash and lipodystrophy which are attributed to most of the HAART medication groups. Protease inhibitors are associated with bleeding events and cholestiasis, and nucleoside reverse transcriptase inhibitors (NRTIs) are linked to bone marrow suppression and lactic acidosis [8]. The spectrum of adverse effects related to HAART in developing countries may differ from that in developed countries because of the high prevalence of conditions such as anemia, malnutrition, and tuberculosis and frequent initial presentation with advanced HIV disease [9].

A review on HAART adherence and interventions reported that HIV stigma and discrimination by friends and family were associated to non adherence. Fear that friends and family might find out HIV status was also one of the reasons for skipping doses. Depression, similar symptoms and anxiety were reported to be associated strongly with non adherence [10]. Another review listed a number of reasons which negatively affect adherence in both developed and developing countries. These included fear of disclosure, forgetfulness, lack of understanding of treatment benefits, complicated regimens as well as being away from medications while medication access and financial problems were more common in the developing countries [11].

On the contrary, lower levels of psychological distress, higher levels of life satisfaction, and higher self-efficacy for adopting medication compliance behaviors were associated with increased adherence. The belief in the medications ability to improve quality of life has also been associated with better adherence [10]. As longevity of PLWHA improves as a result of HAART, the most important question for health professionals and policy makers will be how to maximize quality of life (QoL) [12].

According to the World Health Organization (WHO), “health is a state of complete physical, mental, and social well-being and not merely the absence of disease or infirmity”. This is particularly true for PLWHA because of the chronic and debilitating nature of the illness and the stigma and discrimination [13].

The term QoL has been used to describe the overall sense of well being with respect to happiness and general level of satisfaction with life. It incorporates various domains including health, housing, jobs, schools, the neighborhood, culture, values and spirituality which make it complex to measure [14]. QoL has also been used to describe the extent to which human needs are met or the level of perception of satisfaction or dissatisfaction of individuals or groups in various life domains. It is summarized as an indicator of the extent of fulfillment of objective human needs in relation to personal or group perceptions of subjective well being [15].

Health-related quality of life (HQoL) is a multidimensional component of the patient reported outcome which involves patients evaluation of themselves based on perception of a disease and/or its treatments [16]. HQoL encompasses the aspects of QoL which influence physical or mental health. These include physical and mental health perceptions and various conditions which can affect them. These are health risks and conditions, functional status, social support, and socioeconomic status [14].

Many different instruments have been developed to describe and quantify HQoL. These include HIV-specific instruments such as the medical outcomes study-HIV [17], the HIV overview of problems evaluation system (HOPES) [18] and the WHO Quality of Life instrument module (WHOQOL) for international assessment of HIV/AIDS [19]. The WHOQOL-HIV instrument provides a promising means for quality of life assessment for PLWHA in diverse cultural settings [20].

The WHOQOL-HIV instrument focus on six major domains, referred to as Physical, Psychological, Level of independence, Social, Environmental and Spiritual. Those domains of HQoL are varied in terms of the socio-demographic characteristics and disease related variables. “Socio-demographic characteristics, including age, gender, education, income employment status, and disease related variables such as disease state, opportunistic infection and CD4 count, have been found to be strongly associated with HQoL of PLWHA” [21].

In the effort to document and assess HQoL of PLWHA various studies have been conducted globally and locally using the WHO Quality of Life of HIV specific instrument brief (WHOQOL-HIV BREF). Among these were findings from studies in Bangladesh and Vietnam which reported low to moderate levels of means scores of QoL in the six domains assessed. The relatively highest scores in the cited studies were for the domains of spirituality and environmental health from the Bangladesh and Vietnam studies respectively [22, 23].

Other studies from Africa also reported on the level of QoL of PLWHA on HAART using WHOQOL BREF including findings from Nigeria where occupation, income, education and discrimination were found to be associated with QoL scores for many of the domains. A finding from Zambia reported a high proportion of patients with good or very good levels of quality of life [24, 25].

In Ethiopia few studies have been conducted which assessed QoL among PLWHA focusing on the change in HQoL of patients after initiation of HAART, predictors HQoL of QoL and gender differences in QoL [26–29]. Another study from the northwestern town of Bahir Dar in Ethiopia also reported on QoL of PLWHA which was found to be low in almost all of the domains including general (1.85), physical (2.55), psychological (2.66), social (6.4) and mental (6.4) from a maximum of 8, 24, 32, 16 and 24 respectively [30].

Despite the conduct of numerous studies on HQoL of PLWHA across the world and the availability of measuring instruments, such studies are limited in Ethiopia [31–33]. In addition, assessing HQoL of PLWHA on HAART is important to indicate the status of patients on the therapy. This study, therefore, assessed the perceived HQoL of PLWHA in Gondar University Referral Hospital (GURH) using WHOQOL-HIV BREF. The present study also assessed the individual domain scores and the presence of relationship between those domains and, socio-demographic characteristics and disease variables. It is hoped the result will identify the HQoL domains with poor result and the factors responsible, so that health professionals and policy makers who are involved in the care of PLWHA get informed of the kind of problems that need to be tackled.

## Methods

### Study setting and design

A health facility based cross-sectional study was conducted at GURH, which is located in the northwestern Ethiopian town of Gondar. The institution is a referral and teaching hospital with a catchment population of more than 5 million. GURH is a 400 beds hospital with a range of specialties including pediatrics, surgery, gynecology, internal medicine, HIV care and various others (GURH Statistics and Information Office: Annual Report on Health Services and Employees, Gondar, Ethiopia 2013, unpublished).

### Sampling

The sample size of the participants to be included in this study was calculated using a single proportion formula. In calculating the sample size, a z value of 1.96 as the degree of accuracy at 95% confidence interval, proportion of PLWHA with QOL level of better than average was assumed to be 50% and the margin of error was considered 0.05. Based on this the sample size was calculated to be 385 and with a 5% addition for possible nonresponse the final sample size was 403.

The study population was any PLWHA above 18 years of age and who came to GURH HAART pharmacy for taking HAART during the study period. However, HIV/AIDS patients with incomplete medical chart, mental illness or patients not willing to participate in this study were excluded. Incomplete medical chart refers to the absence of CD4 count or WHO clinical stage of the patients. Psychiatric patients who were not in a position to appropriately respond for the items provided were classified as mentally ill and were excluded.

The study was conducted during the period of April 2014 to May 2014. The total number of active HAART users at GURH was 5394. Considering the 22 weekdays within a study period and a relatively uniform flow of patients across each day, every 14th patient was included in the study. The first client was selected daily through drawing a number from 1 up to 14 and continuing with every 14th number until the daily sample limit was reached. Accordingly, the daily sample limit was 19 patients for 7 days and 18 patients for 15 days.

### Data collection instrument and process

A semi-structured questionnaire containing mostly closed ended and a number of open ended questions was developed by adopting WHOQOL-HIV BREF. The instrument was translated into Amharic language and disease related variables including CD-4 count and WHO clinical stage were added. Hence, it contained socio-demographic, clinical and the six domains of health-related quality of life. The Amharic questionnaire was translated back to English to ensure the translated version gives the proper meaning. The revised Amharic questionnaire was pre-tested using 40 patients and the final version was produced.

Data concerning CD-4 count and the WHO clinical stage of the patients were extracted from their medication record. The data were collected by three final year undergraduate pharmacy students through interviewer-administered questionnaire. The data collection was made between 8:00 AM and 5:00 PM at the HAART pharmacy of GURH for a duration of one month from April 05 to May 04, 2014.

The semi-structured questionnaire consisted of 31 five point Likert scale items, which are grouped into six domains of HQoL; physical health, psychological well-being, level of independence, social relation, environmental health and spiritual health.

The Physical domain describes 4 facets: pain and discomfort, energy and fatigue, sleep and rest and symptoms related to HIV. The Psychological domain describes 5 facets: positive feelings, concentration, self-esteem, bodily image and appearance and negative feelings. The Level of independence domain describes 4 facets: mobility, activities of daily living, dependence on medication and treatment and work capacity. The Social relationships domain describes 4 facets: personal relationships, social support, sexual activity, and social inclusion. The Environment domain describes 8 facets: physical safety and security, home environment, financial resources, health and social care: accessibility and quality, opportunities for acquiring new information and skills, participation in and opportunities for recreation activities, physical environment, transport. The Spiritual domain describes 4 facets: personal beliefs, forgiveness and blame, concerns about the future, death and dying [34].

Individual items are rated on a 5 point Likert scale where 1 indicates low, negative perceptions and 5 indicates high, positive perceptions. Facet scores are the mean of the four items in each facet. Domain scores were computed first by adding the facet in the respective domain, then dividing it by the number of facets in that domain, and eventually by multiplying it with 4. The domain scores were multiplied by 4 so as to be directly comparable with the scores used in WHO quality of life-100 (WHOQOL-100). Accordingly, the score ranged from 4 (worst possible HQoL) to 20 (best possible HQoL) [35].

### Data entry and analysis

The Data collected were entered into and analyzed using Statistical Package for Social Sciences (SPSS) version 20.0 software for Windows. The domains’ mean scores were calculated using WHO user manual on how to score and code WHOQOL-HIV instruments. Domain scores were scaled in a positive direction where higher scores denote higher HQoL. Some items like dependence on medication and death pain were scaled in a negative direction, meaning that for these facets higher scores do not denote higher HQoL. These items were reversed so that high scores reflect better HQoL. Hence, the formula 6-x was used [35]. Cronbach’s alpha coefficient was calculated to determine the internal consistency of the instrument. Independent *t* test was employed to assess the difference in HQoL domain between sex, age, place of residence, current acute illness and CD-4 count. On the other hand, one-way analysis of variance (ANOVA) was used to check the difference among educational status, marital status and WHO clinical stage with regard to domains of HQoL. In doing so the different analysis of 95% CI and *P*-value of less or equal to 0.05 was taken as cutoff value for statistical significance.

## Results

### Socio-demographic characteristics

During the one-month interview period, 468 patients were approached for the participation. 65 of the patients were not willing to participate and 3 questionnaires were discard for the lack of complete medical chart. Of the total of 400 participants, more than half the respondents were female (54.8%). Most of the respondents were above the age of 30 (60.8%) and resided in urban areas (73%). The percentage of the respondents who were married and had finished their secondary education was 42.8% and 35.5% respectively. A clinical stage one (66.8%) was predominant and the majority of the participants (55.5%) had a current CD-4 count less than 500 cells/mm^3^ [Table 1].

**Table 1:** The socio-demographic characteristics of the respondents, GURH 2014

Variables	Frequency (%)
Sex	
Male	181 (45.3)
Female	219 (54.7)
Age	
< 30	157 (39.5)
≥ 30	243 (60.8)
Current CD-4 count (cell/mm^3^)	
< 500	222 (55.5)
≥ 500	178 (44.5)
Marital status	
Single	108 (27.0)
Married	171 (42.8)
Divorced	70 (17.5)
Widowed	51 (12.8)
Residence	
Urban	292 (73.0)
Rural	108 (27.0)
Educational status	
Illiterate	100 (25.0)
Primary	95 (23.7)
Secondary	142 (35.5)
Higher education	63 (15.8)
WHO clinical stage	
Stage 1 (Asymptomatic)	267 (66.8)
Stage 2 (Mild symptoms)	105 (26.3)
Stage 3 (Advanced symptoms)	25 (6.3)
Stage 4 (Severe symptoms)	3 (0.8)

### The overall HQoL

The mean score of HQoL was the highest for the physical domain (15.55), followed by the spirituality domain (15.47), the level of independence domain (15.27), the psychological domain (13.92), the environment domain (12.78) and the social relationship domain (12.11) [Table 2].

**Table 2:** The mean scores of domains of HQoL and overall perception of HQoL, GURH 2014

Domains	Mean (±SD)
Physical health	15.55 (3.04)
Psychological health	13.93 (2.80)
Level of independence	15.27 (3.25)
Social relationship	12.11 (2.85)
Environment health	12.78 (2.33)
Spirituality health	15.47 (3.47)
Overall perception of HQoL	12.57 (2.96)

Cronbach’s alpha was used to determine the internal consistency of the instrument as well as its domains. The Cronbach’s alpha coefficient of physical domain (0.82), spiritual domain (0.89), level of independence domain (0.88), physical domain (0.81), environmental domain (0.85) and social domain (0.79) were adequate.

Patients were asked to rate their perception to overall quality of life, using a scale ranged from very poor (score of 1) to very good (score of 5). Half of patients (50%) rated their overall perceived HQoL as neither good nor poor, by giving a score value of 3. A significantly higher number of patients (32.75%) rated their overall perceived HQoL as good compared to those who rated their overall perceived HQoL as poor (14.5%) or very poor (2.75%). Accordingly, the overall perceived quality of life by PLWHA is 62.55%.

### Difference in HQoL among respondents

The association between the mean scores of HQoL domains and the socio-demographic characteristics and disease related variables was examined. Based on the independent samples *t* test performed on some socio-demographic characteristics and disease related variables, the sex and age of participants were significantly associated with the psychological health. Patients who live in urban areas showed higher level of independence compared to those who live in rural areas. However, the current illness condition and the level of CD-4 of the respondents do not show significant association with any of the six domains of HQoL [Table 3].

**Table 3:** Test of significance of variation (Independent sample *t* test) in HQoL by socio-demographic characteristics and disease variables, GURH 2014

Variables		Physical health	Psychological health	Level of independence	Social relation	Environmental health	Spirituality
Sex	Male	15.392	13.829	14.966	12.237	12.897	15.663
	Female	15.675	14.020	15.516	12.004	12.678	15.310
	T-test	−0.928	−0.676**	−1.686	0.814	0.940	1.011**
Age	<30	15.636	13.966	15.452	12.191	13.130	15.369
	≥30	15.489	13.912	15.148	12.057	12.549	15.535
	T-test	0.472	0.189**	0.914	0.457	2.455	−0.465
Residence	Urban	15.657	14.134	15.411	12.119	12.804	15.575
	Rural	15.250	13.392	14.879	12.083	12.703	15.185
	T-test	1.190	−0.676	1.454*	0.114	0.385	0.998
Current acute illness	Yes	13.9380	12.6822	13.7752	11.2171	12.1473	14.0465
	No	16.3134	14.5403	15.9851	12.5410	13.1119	16.1381
	T-test	−7.7940	−6.4950	−6.6750	−4.4420	−3.9520	−5.8500
CD-4 count	< 500	15.2973	13.7838	14.9414	11.9189	12.4910	15.4279
	≥ 500	15.8596	14.1213	15.6742	12.3483	13.1348	15.5225
	T-test	−1.8420	−1.1990	−2.2520	−1.501	−2.7740	−0.2700

P* < 0.05, P** < 0.01

In one-way ANOVA performed on portions of socio-demographic and disease related variables, the WHO clinical stage showed significantly association with all of the six domains of HQoL. The educational status of the respondents was found to be strongly associated with the social relationship and environmental health. In addition, marital status was significantly associated with the environmental health of the respondents [Table 4].

**Table 4:** Test of significance of variation (one-way ANOVA test) in HQoL by socio-demographic characteristics and disease variables, GURH 2014

Variables		Physical health	Psychological health	Level of independence	Social relation	Environmental health	Spirituality
Educational status	Illiterate	15.040	13.504	14.8900	11.3100	12.0600	15.4800
	Primary	15.600	14.012	15.1789	12.2211	12.3737	15.4947
	Secondary	15.704	14.090	15.5915	12.4155	13.1268	15.3662
	Higher education	15.920	14.1460	15.2698	12.5238	13.7381	15.6208
	F-test	1.382	1.0790	0.9430	3.7430**	9.3200**	0.1000
Marital status	Single	15.4537	13.9037	15.1204	12.4259	13.1435	15.3333
	Married	15.5263	13.8012	15.2164	11.8947	12.4064	15.5848
	Divorced	15.7143	14.1829	15.4857	12.0857	12.8786	15.4000
	Widowed	15.5882	14.1020	15.4510	12.1961	13.1078	15.4706
	F-test	0.1090	0.3760	0.2460	0.7850	2.764*	0.1270
WHO clinical stage	Stage-1	15.9401	14.2772	15.8090	12.4607	13.1610	15.9288
	Stage-2	14.7619	13.2800	14.0381	11.5048	12.0429	14.5333
	Stage-3	14.6400	13.2480	14.8000	10.8800	11.9800	14.5200
	Stage-4	15.6667	12.0000	14.0000	12.3333	11.0000	15.3333
	F-test	4.6850**	4.3300**	8.2280**	4.6140**	7.8570**	4.8650**

P* < 0.05, P** < 0.01

## Discussion

The study assessed the HQoL of PLWHA who came to receive HAART from the HAART pharmacy of GURH. In this study, HIV/AIDS was prevalent in patients with secondary educational status (35.5%). Only 15.8% of the respondents have attended higher education studies, a status which might increase the awareness and method of prevention against HIV/AIDS. A study in Brazil also showed that 8 years of education is associated with better HQoL [36].

Some studies have documented low performance for women in some aspects of HQoL [37] while in some other studies had shown the opposite [22]. In this study, female respondents showed a significantly higher psychological health than men. This can be attributed to many factors including, but not limited to, increased fertility desire of women following the use of HAART [7].

Urban residents showed higher values in all domains of HQoL than PLWHA in rural areas. A relatively better financial status, infrastructures and increased support for patients in urban areas might be some of the contributing factors for this difference. Among the domains urban residents recorded a statistically significant higher score in terms of level of independence which could be attributed to the relatively less physically demanding activities in urban areas compared to farming in rural areas.

All the domains of HQoL showed a higher mean scores than a study in Bangladesh, the only exception being the social relationship domain. The mean score of the social relation domain (12.11) was lower than the Bangladesh study (12.98). The reason behind this may be due to the different study design used or a difference in socio-demographic characteristics or the presence a relatively more stigma and discrimination in Ethiopia which had been and continues to be a serious problem associated to the disease [22, 38]. The domain mean scores of this study and a study in Bangladesh were much higher than those reported in the studies in Bahir Dar and Iran [22, 26, 37]. The results from the Bahir dar study, however, cannot be compared directely to this study as different data collection tools were used. The perceived overall quality of life (12.57) found in this study can be considered as moderate using a median of HQoL mean scores (a value of 10) as a cut-off point.

The mean scores of social relationship and environmental health were lower than any other aspects of HQoL. A study conducted in Nigeria [39] also reported that PLWHA had a lower quality in the social relationships and environment domain. Those results may be related to discrimination as well as poor living conditions in their physical environment. The psychological health (13.93) was found to be lower than physical health, level of independence and spirituality. This result is an indicative of signifying the need to combine HAART with psychological interventions.

In this study, WHO clinical stage showed a significant association with all domains of HQoL. A similar relation is reported in the Bangladesh study, which found a significant association of WHO clinical stage with the physical health, level of independence, perception of HQoL and perception of overall health [22]. The higher WHO clinical stages may be associated to limited day-to-day activities, which may eventually results in poor HQoL.

A higher CD-4 count was reported to be associated with better HQoL, especially with regard to physical domain [7]. In this study, however, CD-4 count didn’t not show a statistically significant association with any of the domains of HQoL. Moreover, a similar no association was reported in the study conducted in Bahir Dar [30].

In this study, current acute illness was found to bear no significant relation with all domains of HQoL. However, Socio-demographic characteristics like age, residence, educational status and marital status showed a significant association with one or more domains of HQoL. The study from Bangladesh also reported a significant association of age and place of residence with one or more of domains of HQoL. The Bahir dar and the Iran studies, however, found no significant association between educational status or marital status and any domains of HQoL [22, 30, 37].

This study has managed to add to the scarce literature in the area of quality of life of HIV/AIDS patients in Ethiopia and can be helpful in showing points of intervention to stakeholders to intervene toward improving the life of PLWHA.

### Limitations

The study had some limitations as it included only patient taking HAART. Inclusion of HIV patients who did not start HAART would have given a more comprehensive result. Researches with a different study design, including qualitative methods, can overcome this limitation.

## Conclusions

The six domains of HQoL were found to be moderate. The physical health and spirituality of the patients were relatively higher than their social relationship. Sex, age, educational status, residence and marital status were significantly associated with at least one domain of HQoL. Moreover, WHO clinical stage was strongly associated with all HQoL domains.

Strategies to improve psychological supports and environmental health, and strengthening social relations seems relatively more demanding than other domains of HQoL. Primary health care support can be used to improve the psychological and environmental health of PLWHA. In this aspect health care providers can use the findings to focus their interventions to address the social aspects of the problems of their patients in addition to the clinical aspects. Social sensitization to avoid stigma and discrimination can improve those domains of HQoL since it may help to create a sense of safe living conditions or help PLWHA to receive a necessary support from their friends.

## Abbreviations

ANOVA: Analysis of variance
CI: Confidence interval
GURH: Gondar University Referral Hospital
HAART: Highly active anti-retroviral therapy
HOPES: HIV overview of problems evaluation system
HQoL: Health related quality of life
PLWHA: People Living With HIV/AIDS
SPSS: Statistical Packages for Social Sciences
WHO: World Health Organization
WHOQOL BREF: World Health Organization quality of life of HIV specific instrument brief
